# Mentalization-based treatment in groups for adolescents with borderline personality disorder (BPD) or subthreshold BPD versus treatment as usual (M-GAB): study protocol for a randomized controlled trial

**DOI:** 10.1186/s13063-016-1431-0

**Published:** 2016-07-12

**Authors:** Emma Beck, Sune Bo, Matthias Gondan, Stig Poulsen, Liselotte Pedersen, Jesper Pedersen, Erik Simonsen

**Affiliations:** Child and Adolescent Psychiatric Department, Region Zealand, Smedegade 16, 4000 Roskilde, Denmark; Psychiatric Research Unit, Region Zealand, Fælledvej 6, 4200 Slagelse, Denmark; Department of Psychology, University of Copenhagen, Øster Farimagsgade 2A, 1353 Copenhagen K, Denmark; Institute of Clinical Medicine, Faculty of Health and Medical Sciences, University of Copenhagen, Blegdamsvej 9, 2100 Copenhagen, Denmark

**Keywords:** Mentalization-based treatment, Adolescence, Borderline personality disorder, Group psychotherapy, Mentalizing

## Abstract

**Background:**

Evidence-based outpatient psychotherapeutic programs are first-line treatment of borderline personality disorder (BPD). Early and effective treatment of BPD is crucial to the prevention of its individual, psychosocial, and economic consequences. However, in spite of recent advantages in diagnosing adolescent BPD, there is a lack of cost-effective evidence-based treatment programs for adolescents. Mentalization-based treatment is an evidence-based program for BPD, originally developed for adults.

**Methods/Design:**

Aims/hypotheses: We will investigate whether a specifically designed mentalization-based treatment in groups is an efficacious treatment for adolescents with BPD or subthreshold BPD compared to treatment as usual. The trial is a four-center, two-armed, parallel-group, assessor-blinded randomized clinical superiority trial. One hundred twelve patients aged 14 to 17 referred to Child and Adolescent Psychiatric Clinics in Region Zealand are randomized to 1 year of either mentalization-based treatment in groups or treatment as usual. Patients will be included if they meet at least four DSM-5 criteria for BPD. The primary outcome is self-reported borderline features at discharge. Secondary outcomes will include self-harm, depression, BPD criteria, externalizing and internalizing symptoms, and social functioning, together with parental reports on borderline features, externalizing and internalizing symptoms. Measures of attachment and mentalization will be included as mediational variables. Follow-up assessment will take place at 3 and 12 months after end of treatment.

**Discussion:**

This is the first randomized controlled trial to test the efficacy of a group-based mentalization-based treatment for adolescents with BPD or subthreshold BPD. If the results confirm our hypothesis, this trial will add to the treatment options of cost-effective treatment of adolescent BPD.

**Trial registration:**

Clinicaltrials.gov NCT02068326, February 19, 2014.

**Electronic supplementary material:**

The online version of this article (doi:10.1186/s13063-016-1431-0) contains supplementary material, which is available to authorized users.

## Background

Borderline personality disorder (BPD) is a severe mental disorder with symptoms such as affective instability, impulsivity and self-harm [1]. In comparison to other personality disorders (PDs), BPD is associated with more severe impairments in social functioning [2, 3], higher rates of psychiatric comorbidity and increased risk of suicide attempts [4]. Prevalence in the general population ranges from 0.5 to 2.7 % [5–9]. In clinical populations the prevalence ranges between 9 and 22 % in outpatient settings [10, 11], and up to 40 % in inpatient settings [12]. Research into the etiology of BPD indicates that it is multifactorial and includes genetic dispositions, neuropsychological dysfunctions, and environmental factors such as neglect and physical abuse [13].

It has been disputed whether it is possible and meaningful to diagnose PDs in general and BPD in particular in adolescents [14–16]. However, there is evidence that BPD can be diagnosed reliably and validly in adolescents with reliability rates comparable to those found with adults [17–22]. The possibility of diagnosing adolescent PD has been introduced in the *Diagnostic and Statistical Manual of Mental Disorders, fifth edition* (DSM-5) [1] and BPD prevalence rates among adolescent inpatients are similar to those of adult inpatients [23, 24]. Stability of PD diagnoses among adolescents also resembles that of adults [25].

BPD is associated with both chronic medical illnesses and comorbid psychiatric disorders, and psychiatric comorbidity may be even higher in adolescents than in adults [26–31]. Patients with BPD tend to use inpatient and outpatient treatment far more than patients with other PDs [32] leading BPD to be associated with costly health service use [27, 33], also in adolescence [34, 35]. Therefore, the development of early and effective treatment programs, which include adolescents with BPD at the threshold level is important and may have long-term benefits for patients, their families and society [36–40]. While pharmacological treatments can reduce specific symptoms experienced by BPD patients, they fail to bring about a fundamental or long-lasting change in the disorder itself. In contrast, several structured outpatient psychotherapeutic programs such as dialectical behavior therapy (DBT), mentalization-based treatment (MBT), transference-focused therapy (TFP), cognitive behavioral therapy (CBT) and schema-focused group therapy (SFT) have been shown to be efficacious. Although further evidence is still warranted, psychotherapy is suggested as the primary treatment for BPD [41, 42].

Adaptation of structured and manualized treatment programs to adolescents with BPD is novel, and relatively few controlled studies have tested their efficacy: In an randomized controlled trial (RCT) by Mehlum et al., DBT-A was found to be superior to the control condition in reducing the frequency of self-harm, both at treatment end and at follow-up [43, 44]. Other studies on feasibility and efficacy of DBT-A also indicate a positive outcome for DBT-A [45–49], but these studies are uncontrolled, small scale, and use different outcome measures, leading the evidence base to be considered insufficient [50]. As the target population for DBT-A is adolescents with suicidal and self-harming behavior, the overlap between this group and adolescents with BPD is only partial [50], and the efficacy of DBT-A for treating BPD in adolescents is unknown. Chanen et al. [51] tested individual cognitive analytic therapy (CAT) for adolescents with BPD symptoms and found no significant differences between the outcomes of CAT and the control condition at 24 months, but with faster rates of symptomatic improvement in the CAT condition. Schuppert et al. [52] also found no superiority of a 17-session group-based emotion regulation training course. In contrast, Rossouw and Fonagy’s [53] adaptation of mentalization-based treatment (MBT) for borderline personality patients to adolescents yielded more promising results: A 1-year treatment program for adolescents with self-harming behavior (of whom 73 % met the diagnostic criteria for BPD) comprising weekly individual MBT sessions and monthly family-therapy sessions, was more effective than treatment as usual (TAU). In an uncontrolled study, Laurenssen et al. [54] adapted MBT to an inpatient setting with adolescent BPD patients, and found a significant improvement in Axis-I symptomatology and personality functioning.

MBT is specifically developed to treat BPD and its efficacy of treating BPD in adults has been tested in several RCTs [55–58]. MBT is based on attachment theory and psychodynamic principles and has a high degree of structure and a clear treatment goal of improving patients’ mentalizing skills [55–59]. Mentalization has been defined as the capacity to understand and interpret - implicitly and explicitly - one’s own and others’ behavior as an expression of mental states such as feelings, thoughts, fantasies, beliefs, and desires [60]. The capacity develops during childhood and is intimately linked with the quality of early attachment relationships [61]. When the caregivers affective mirroring of the child’s mental state is marked and congruent, i.e., is representative of the child’s mental state, and not her own, it serves as a representation of the state that the child can incorporate into its own representation of self [60]. The theoretical framework of MBT links BPD pathology to an impairment in the capacity to mentalize. In states of emotional arousal, BPD patients are vulnerable to shifting toward a non-mentalizing mode. In short, MBT offers therapeutic techniques for identifying when this shift is occurring and how to help the patient return to a mentalizing mode, where an understanding of one’s own and others’ minds can be used to regulate the patient’s emotional state [40]. In the original model for MBT, group psychotherapy is supported by individual psychotherapy that ensures patients’ attendance to group sessions by motivating and working constructively with patients’ negative experiences that could otherwise lead them to drop out of treatment [59]. However, such a relatively resource-demanding treatment model can impede implementation, and research into the effectiveness of the separate modalities of MBT is sparse.

While the overall aim of developing patients’ mentalizing skills overlaps in individual and group therapy, there may be some particular advantages to using the group modality in the treatment of BPD. Karterud [62] summarizes the literature on these advantages as follows: First, the relatively intimate two-person relationship of individual therapy is likely to activate attachment patterns and transference/countertransference that are emotionally too burdening for the BPD patient to endure. In the group, such interpersonal processes will be “spread out” on different group members and therefore be experienced as less intense. Second, as the interpersonal difficulties associated with BPD naturally unfold between group members, group therapy offers an opportunity to explore and work on them in vivo. Third, difficulty with authority figures experienced by many BPD patients can diminish their receptiveness to feedback coming from the therapist. In contrast, when feedback comes from group members, this difficulty may be bypassed. Furthermore, compared to individual therapy, group therapy is considerably less expensive.

Including parents in the MBT program for adolescent BPD and targeting the parent-child relationship is important. Parental and family circumstances are contributing factors to the development of BPD [63, 64], and BPD symptoms such as affect dysregulation and impulsivity are especially prone to be exhibited within the context of attachment relationships [40], such as the relationship between adolescents with BPD and their parents. We suggest that guidance for parents on how to understand and respond to their child in interpersonal stressful situations can promote mentalization and co-regulation within the parent-adolescent relationship, and that these intersubjective experiences may serve as a pathway to development of the adolescents’ mentalizing skills. We also propose that it will reduce premature dropout to educate and guide the parents about the importance of supporting their child in times of low motivation for attending treatment sessions [65].

To our knowledge, group-based MBT for adolescent BPD or subthreshold BPD that includes parents in the treatment has not yet been tested for efficacy in a controlled study. However, in an uncontrolled feasibility trial (N = 25) of a group-based MBT program similar to the one presented in the present protocol, we found symptomatic improvement in 92 % of the patients. On the Borderline Personality Features Scale for Children (BPFS-C), which is also the primary outcome measure in the present trial, the difference in before and after scores were highly significant (Bo, Sharp, Beck, Pedersen, Gondan and Simonsen (submitted)).

## Aims and hypotheses

We will investigate whether a specifically designed treatment program, Mentalization-based treatment in groups for adolescents with BPD or subthreshold BPD, is an efficacious treatment compared to treatment as usual (TAU). The MBT program includes an introduction to mentalization (MBT-I), mentalization-based group therapy (MBT-G) and mentalization-based psychoeducation for the parents of the patients (MBT-P) [62, 66]. The present study will test if this specifically designed group-based MBT program is superior to TAU as measured by a decrease in borderline personality features after the last MBT-G session (session no. 40).

## Methods/Design

The study is a randomized two-armed, parallel group, assessor-blinded continuous outcome superiority trial, comparing a group-based MBT program with TAU in 112 adolescents with BPD or subthreshold BPD, following intention-to-treat (ITT) principles. The study will be carried out at four outpatient child and adolescent psychiatric clinics within The Child and Adolescent Psychiatric Department, Region Zealand, Denmark.

### Eligibility criteria

#### Inclusion criteria

1. Age from 14 to 17 years
2. Meeting a minimum of four DSM-5 BPD criteria
3. Total score higher than clinical cutoff (67) on the Borderline Personality Features Scale for Children (BPFS-C)
4. Parents’ or parent substitutes’ commitment to participate in the MBT-P program and to support their child’s participation in the MBT-I and MBT-G program
5. Written informed consent

#### Exclusion criteria

1. Comorbid diagnosis of pervasive developmental disorder, learning disability (IQ < 75), anorexia, current psychosis, diagnosis of schizophrenia or schizotypal personality disorder and antisocial personality disorder (DSM-5)
2. Any other mental disorder other than BPD considered the primary diagnosis
3. Current (past 2 months) substance dependence (but not substance abuse)
4. Current psychiatric inpatient treatment
5. Receiving any other psychotherapeutic treatment
6. Not living with parent(s) or parent’s substitute(s), who are able to participate in the MBT-P program
7. Not fluent in Danish

### Recruitment and baseline procedures

**Fig. 1:**
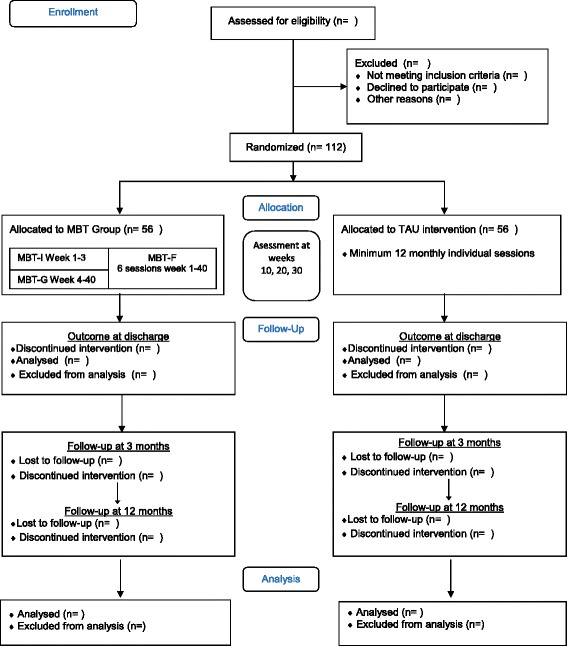
Flow diagram of M-GAB study design. Consort flow diagram

Participants from child and adolescent psychiatric in- and outpatient clinics in Region Zealand will be screened for eligibility as part of their psychiatric review and referred to further assessment, provided they do not meet any exclusion criteria. A simple screening instrument has been developed, and staff will be trained in using it prior to recruitment. When clinicians encounter patients who fulfil the inclusion criteria, the patients are sent to an assessor, who will inform the patient and parents thoroughly about participation in the M-GAB trial including the assessment procedure. Patients are then invited to two sessions of assessment for eligibility. Both sessions last between 2 and 3 hours including breaks. Subsequently the family is told if they are invited to participate in the randomization, and if so, outcome measures at baseline are administered. Randomization will take place after completion of the baseline assessment and patients will be allocated to either the MBT in groups or the TAU group (see below). The flowchart and measurement points are presented in Fig. 1.

### Interventions

Both treatment conditions, MBT in groups and TAU, will be delivered at all four child and adolescent psychiatric clinics in Region Zealand.

### Experimental intervention: MBT in groups

Mentalization-based group therapy is constituted by a set of treatment principles. Naturally, some principles overlaps with individual MBT (i.e., “regulating arousal” and “keeping an affect focus”), but others are specific to the group component (i.e., identifying and mentalizing events in the group) [62, 67]. The patients’ profound difficulties with interpersonal functioning and affect regulation require therapeutic techniques potent enough to counteract the difficulties that can arise during sessions. A main therapeutic goal is to balance the management of therapist authority and structure of each session with the open-minded, exploring, and curious stance in order “to create and sustain a mentalizing discourse in the group” [62, 67].

MBT in groups is a 1-year psychotherapy program with three components, MBT-Introduction (MBT-I), MBT-Group (MBT-G) and MBT-Parents (MBT-P).

MBT-I is a structured 3-week introductory psychoeducative program for patients covering the concepts of mentalizing and attachment, and information about BPD. MBT-I includes role plays and discussions of cases and video clips in order to encourage active participation. Furthermore, the group members will be encouraged to participate in small homework assignments between the sessions. MBT-I was developed by Karterud and Bateman [66], and modified for our purpose in collaboration with the authors.

Mentalization-based group therapy (MBT-G) consists of 37 weekly sessions of mentalization-based psychotherapy in groups. Sessions are not accompanied by continuing individual sessions. However, case formulations are made with patients during individual sessions with both therapists, and this process scaffolds and supports the therapeutic work in the group. The overall purpose of the case formulation sessions is for therapists and patient to develop a clear mutual understanding of the patient’s main difficulties and psychotherapeutic focus points, which can be drawn on during group sessions. The case formulation is to be formulated within the mentalization-based framework, applying the theoretical understandings and principles from mentalization theory. The individual focus in these sessions organizes thinking for therapists and patient and supports the mentalizing work in the ensuing group sessions [62]. Three individual case formulation sessions are scheduled before the initiation of the MBT-G. The patient will also be informed which therapist is his or her contact person, and an updated crisis plan with the therapist’s contact details is included in the formulation. After eight to ten group sessions and again toward the end of the program (after 25 group sessions), a case formulation session is scheduled for revising the formulation according to the deepened knowledge and development of the patient.

MBT-P is a psychoeducation program for the patients’ parents running parallel to MBT-I and MBT-G. It is a slow-open six-session program comprising role plays, plenary analysis of difficult interpersonal events between parent and adolescent and therapists’ presentations with information on BPD, mentalization, and attachment. The importance of parents’ continuous support of their adolescents’ attendance to treatment is also discussed. All sessions have a length of 90 minutes. MBT-P is adapted from Karterud and Bateman’s manual [66]. An overview of the entire MBT in groups program is presented in Fig. 1. Both MBT-P as well as the contact-person function mentioned above are intended to prevent premature dropout of the treatment.

#### Therapists

MBT will be delivered by clinical psychologists and a psychiatrist. Prior to starting the MBT treatment, therapists will have participated in a 2-day introduction to MBT theory and basic principles and a 5-day training program by Professor Sigmund Karterud, who developed the manual for MBT-G and MBT-I in collaboration with Anthony Bateman [62, 66]. Replacement of therapists during the trial may be necessary, in which case at least one therapist in each group will have finished training, while the co-therapist is undertaking or waiting to begin training. National specialists in MBT and adolescent psychiatry provide 1–2 hours of supervision per month to secure adherence to the manual. All sessions are videotaped and 10 % are randomly selected for ratings of adherence to the treatment manual applying the MBT-G adherence and competence scale [62]. Therapists delivering MBT within this trial will not deliver TAU, but may carry out non-MBT-based clinical work outside the trial such as clinical assessments or case management for in-patients.

#### Organization of MBT in groups within the four clinics

Whenever patients terminate/drop out of a MBT-G group and new patients can be admitted into the group, a new MBT-I group starts up. As a default, patients will be offered MBT-I in their local clinic, but may also choose to enter MBT-I in a different clinic in order to participate in a larger group. MBT-I groups starting up with less than five patients are run by one therapist. Groups with five or more patients are delivered by two therapists, Patients start MBT-G treatment immediately after completion of MBT-I. The MBT-G treatment will run in five slow-open groups across the four clinics with a maximum of eight patients in each.

### Treatment as usual

TAU is based on supportive techniques and comprises psychoeducation, counseling and, if needed, ad hoc crisis management with the overall aim of alleviating BPD symptomatology. TAU is delivered by child and adolescent mental health professionals with thorough experience in working with psychopathology in adolescents. They will be psychologists, psychiatrists, social workers or nurses employed in child and adolescent psychiatric clinics in Region Zealand. Adherence to the supportive approach is monitored in regular supervision, as TAU is not manualized. For the purpose of this study, we defined a limit for the minimum treatment by standardizing to *at least* 12 individual monthly sessions. Additional contact may vary across clinics and according to the needs of the patient. The supervisors and staff selected to carry out the TAU treatment and supervision have no previous MBT training. They will not be undertaking MBT training during the trial, nor will they deliver MBT to patients in- or outside the trial. We will monitor and register both the MBT and TAU patients’ contact to the health system during the treatment period, to be able to report both quantitatively and qualitatively the treatment both groups have received.

### Medication in the experimental intervention group and in TAU

All patients will continuously be monitored for their use of medication during the psychotherapeutic intervention, and any differences between the groups will be analyzed as a possible confounder for outcome.

A protocol containing guidelines for pharmacological treatment is provided (available on request) and medication will be registered. The protocol will follow the national recommendations for treating mental disorders in adolescents, and more specifically the new guidelines released in June 2015 from the National Board of Health for treatment of borderline personality [68]. All psychiatrists responsible for medical treatment will be committed to this medical protocol adherence.

### Assessment and instruments

#### Diagnostic evaluation at baseline

Baseline assessment will be carried out prior to randomization by the first author and a research assistant, both of whom are clinical psychologists. The first author will not carry out treatment in any of the treatment arms. The research assistant may deliver MBT-I to smaller groups of patients. He or she will not deliver TAU.

*Clinical syndromes:* general psychopathology will be assessed with the Mini-International Neuropsychiatric Interview for children and adolescents (MINI-KID 6.0) [69], which is a structured diagnostic interview for children aged between 6 and 17 years old based on 24 DSM-IV and ICD-10 criteria. The MINI-KID generates reliable and valid psychiatric diagnoses for children and adolescents [70].

*Personality disorders* will be assessed with the Structured Clinical Interview for DSM-IV-Axis II (SCID-II) [71]. SCID-II assesses the presence of personality disorders listed in the DSM-5. The SCID-II is considered the “gold-standard” assessment instrument for personality disorders [72], and has shown good psychometric properties [71]. All SCID-II interviews will be carried out by the first author who is a trained and experienced SCID-II interviewer.

*Borderline personality disorder.* Zanarini et al.’s [73] Childhood Interview for DSM-IV Borderline Personality Disorder (CI-BPD) was developed to also allow the identification of subthreshold borderline pathology in children. The CI-BPD is a semi-structured interview in which the interviewer asks a series of questions and subsequently rates each DSM-based criterion on a score of 0 (absent), 1 (probably present) or 2 (definitely present). The CI-BPD has excellent psychometric properties [73, 74] and has demonstrated significant, albeit moderate, agreement to clinician diagnosis at time of discharge in a sample of inpatients (kappa = .47) and good internal consistency with a Cronbach’s alpha of 0.82 [75]. The first author has been trained in the CI-BPD by Zanarini in person and will train all other assessors in the study to secure good inter-rater reliability.

### Outcomes

#### Primary outcome

The primary outcome, the Borderline Personality Features Scale for Children (BPFS-C) [76], is a 24-item dimensional measure adapted from the borderline scale of the Personality Assessment Inventory (PAI) [77] for use with children and adolescents from 9 years of age. It has the same four subscales as the PAI borderline scale: Negative Relationships, Affective Instability, Self-harm and Identity Problems. The 24 items are rated on a 5-point Likert scale, ranging from 1 (not at all true) to 5 (always true) with a higher total score indicating more severe levels of borderline personality features. Crick et al. [76] established evidence for the construct validity and demonstrated high internal consistency. Evidence for cross-informant concordance, criterion and concurrent validity were established by Sharp and colleagues [78]. Satisfactory construct and criterion validity has also been found for the abbreviated 11-item version of the BPFS-C [79]. In an RCT of individual MBT for adolescents [53], the BPFS-C was found to be sensitive to clinical change.

#### Secondary outcome measures

*Depression* will be measured with the Beck’s Depression Inventory for Youth (BDI-Y) from Becks Youth Inventories of emotional and social impairment, which have also proven valid and reliable in Danish [80].

*Self-harm* will be measured using the self-report scale on self-harm from the Risk-Taking and Self-Harm Inventory for adolescents (RTSHIA) [81]. The RTSHIA is a 38-item measure adapted from the adult Self-Harm Inventory [82] to be suitable for use with adolescents. The 38 items are rated on a 4-point Likert scale with higher scores indicating a higher frequency with which the adolescent has engaged in risk-taking or self-harming behaviors. The RTSHIA has been shown to have very high reliability (Cronbach’s alpha = 0.89, test–retest reliability = 0.93) and validity.

*Externalizing and internalizing symptoms* are measured by the Youth Self-Report (YSR) [83] and a corresponding parents’ version, the Child Behavior Checklist (CBCL) [84]. One hundred twelve items are rated on a 3-point scale with “0” for not true, “1” for somewhat or sometimes true, or “2” for very or often true. Both the internalizing and the externalizing scales comprise symptom scales corresponding to DSM diagnoses. A total problem score is derived by summing up all the symptom scales. The YSR and the CBCL are established evidence-based assessment instruments [85], which have also been validated and standardized in a Danish sample [86, 87].

*Borderline personality disorder symptoms* are measured with the Zanarini Rating Scale for Borderline Personality Disorder (ZAN-BPD) [88], which is a semi-structured interview with ratings from 0 (no symptoms) to 4 (severe symptoms) on each of nine items corresponding to the nine DSM-IV criteria for borderline personality disorder. ZAN-BPD has been used in previous studies of pharmacological and psychological treatments for people with BPD. It is reliable, sensitive to change, and has highly convergent validity with structured clinical ratings of BPD [89].

*Global assessment of functioning* will be assessed with the Children's Global Assessment Scale (C-GAS) that has shown validity in measuring functioning in children [90] and sufficient inter-rater reliability in a Danish sample [91].

*Parental reports.* In addition to the parents’ version of the YSR, the CBCL, described above, we will also collect parental reports on borderline personality features using the parents’ version of the BPFS-C, the BPFS-P [78].

*Sociodemographic information* will also be collected at baseline during assessment procedures and from medical records.

Furthermore, reduction in the number of patients’ hospital admissions and visits to the emergency room will be used as a secondary outcome measure. All patients included in the study will be followed in the National Health Register, and we will extract data from the register dating back to the date before the patients were enrolled in the study as well.

### Mediational variables

*Attachment* will be assessed with the Experience of Close Relationships Inventory (ECR) [92] and with the Inventory of Parent and Peer Attachment-Revised (IPPA-R) [93]. The ECR is a 36-item self-report questionnaire measuring attachment in romantic relationships. It has displayed good psychometric properties. The IPPA-R is a valid and reliable 53-item measure of attachment in adolescence. It comprises two scales that measure attachment to parents and peers, respectively.

*Mentalization* will be assessed with the 46-item measure Reflective Function Questionnaire for Youth (RFQ-Y) [94].

### Measurement intervals

The primary and all self-report-based secondary outcomes are measured at baseline, week 10, 20, 30, and after the 40^th^ and final MBT group session. Secondary outcomes based on interviews and expert ratings (the ZAN-BPD, C-GAS) are measured at baseline and discharge. The test-battery designed for intake, including basic clinical evaluation, mediational and outcome measures will take an estimated time of 6 hours per patient. Table 1 lists the primary and secondary outcomes, along with mediational measures.

**Table 1:** Assessments administered at baseline and each follow-up point throughout the trial

Assessment points	Outcome and mediational self-report measures	Expert ratings and clinician-administered measures
Baseline	Patient: BPFS-C, YSR, BDI-Y RTSHIA, ECR-R, IPPA-R, RFQ-Y Parent: CBCL, BPFS-P	Patient: C-GAS, ZAN-BPD
10 weeks, 20 weeks, 30 weeks	Patient: BPFS-C, YSR, BDI-Y RTSHIA, ECR-R, IPPA-R, RFQ-Y Parent: CBCL, BPFS-P	
Discharge	Patient: BPFS-C, YSR, BDI-Y, RTSHIA, ECR-R, IPPA-R, RFQ-Y. Parent: CBCL, BPFS-P	Patient: C-GAS, ZAN-BPD

*BPFS-C* Borderline Personality Features Scale for Children, *YSR* Youth Self-Report, *BDI-Y* Becks Depression Inventory for Youth, *RTSHIA* Risk-Taking and Self-Harm Inventory for adolescents, *ECR-R* Experience of Close Relationships Inventory-Revised, *IPPA-R* Inventory of Parent and Peer Attachment–Revised, *RFQ-Y* Reflective Function Questionnaire for Youth, *C-GAS* Children's Global Assessment Scale, *ZAN-BPD* Zanarini Rating Scale for Borderline Personality Disorder

We will have weekly self-report-based assessment in relation to group sessions (40 measurements) and patients will fill out questionnaires in the beginning of each group session. The assessment comprises a short form of the BPFS-C, the BPFS-C-11, and the short version of the Youth Self-Report, the BPM-Y (19 items). This is to be able to monitor closely when in the treatment program possible progress, and development in the patients’ psychopathology is obtained.

All applied instruments will be applied in Danish translated versions. Published Danish versions exist of the SCID-II, the CBCL, the YSR, the BDI-Y, the ECR-R and the ZAN-BPD. The authors translated the remaining instruments.

### Deterioration

Any patient whose mental health deteriorates during treatment will be taken care of according to official guidelines and treatment recommendations in the Department. We will perform subgroup analyses of patients with an increased score on the primary outcome at discharge (i.e., after the 40th session), in order to enhance our knowledge about what characterizes patients who do not benefit or deteriorate from treatment [95].

### Sample size

Sample size is determined for the primary outcome, namely, the total score of the BPFS-C. A 12-point difference between the two treatment groups is considered to be clinically important. The only outcome study using the BPFS-C to date reported a standard deviation of outcome of 15.4 for psychotherapeutic treatment with a patient group similar to ours. With 90 % power and a two-tailed significance level of 5 %, 72 patients would need to be randomized to the two treatment arms. This calculation does not, however, take into account the similarity of patients who are treated by the same therapists and potential dropout.

Patients in the MBT group will be treated in five groups with two therapists in each group. These are slow open groups with six to seven patients, which will include a new patient as soon as another patient has finished treatment. Group treatments are known to cause similarity of the outcomes within groups, which needs to be accounted for in the sample size planning. We expect an intraclass correlation of 0.03, and a total number of eight patients treated in each of the groups, the design effect is 1.24. Thus, the sample size has to be increased by 24 % (which would be 90 patients). For simplicity, we assume the same intraclass correlation in the control group because the control patients are nested within therapists even if the treatment is individual.

Intention-to-treat (ITT) analysis will be used, as it is the recommended approach to evaluating RCTs ITT analysis includes every subject who is randomized according to randomized treatment assignment and it ignores noncompliance, protocol deviations, withdrawal, and anything that happens after randomization. Hence, ITT analysis preserves prognostic balance produced from the original random treatment allocation, but the treatment effect generated from ITT analysis is generally conservative. We will recruit 20 % more patients to have powerful tests in both the ITT as well as in the per protocol population even in the presence of dropout. This yields a total of 112 patients to be randomized, in a 1:1 ratio.

### Randomization, methods to minimize bias, and blinding

In order to keep assessors blind to the patients’ treatment allocation, all pretreatment assessments will be carried out prior to randomization. Randomization to either MBT or TAU are done online by the trial coordinator (the first author) using a stratified block randomization procedure with a computer-generated allocation sequence with a varying block size kept unknown to the investigators by Public Health and Quality Improvement Data Management*.* Stratification will be according to clinic affiliation and borderline severity, that is, scores on the BPFS-C. More stratification variables are relevant (i.e., socioeconomic status), but not possible to apply due to the small number of participants. Patients randomized to the MBT will initiate the individual case formulation sessions (see Fig. 1) prior to MBT-I, and thereby will initiate the treatment program immediately. Patients randomized to TAU are allocated to a therapist and begin individual sessions immediately.

Assessments during the treatment phase are limited to self-report measures, hence no blinding will be possible. All information given to the participants before completing the self-reports will be standardized.

Regarding outcome assessments at discharge, we will minimize bias that knowledge of treatment allocation could cause by implementing the following strategies: (a) outcome assessors will be blind to treatment allocation, (b) outcome assessors and therapists will not directly communicate with each other, and (c) patients are asked not to reveal their treatment allocation during outcome assessments. Furthermore, outcome assessors will be asked to guess the patients’ treatment allocation so that the effects of possible bias can be examined in the analysis and all outcome assessor interviews will be recorded and a random sample will be re-rated by independent raters. Outcome assessors will be research assistants or clinical psychologists, who have not been trained in MBT, have not been involved in delivering treatment to participating patients, and have not carried out any intake procedures, including baseline assessments. The statistician will perform statistical analyses with the two intervention groups coded as ‘A’ and ‘B’, with randomly chosen therapist identifications.

### Data management and statistical approach

Data-management will be handled by Public Health and Quality Improvement Data Management from Central Denmark Region, Aarhus. They will provide support with randomization, set up questionnaires electronically and will keep the data on their secure servers. Hence, none of the staff members involved in the study will have access to outcome data during the treatment phase.

All analyses will be conducted according to the ITT principle. Characteristics of the treatment groups will be described at baseline. Preliminary analysis will investigate the pattern of missingness at follow-up, and multiple imputation will be used for missing values. The primary outcome is the total score of the BPFS-C, which is treated as a continuous, normally distributed variable. The primary efficacy hypothesis will be tested using a multilevel two-group comparison (MBT vs. TAU), with Group as a main effect, Therapy Group as a random intercept (within the MBT group, see, e.g., [96]), the prerandomization BPFS-C as a continuous covariate and the stratification factor clinic as categorical covariate. The test will be performed at the 5 % two-tailed significance level. The primary test for efficacy will be based on the intention-to-treat population, with all randomized patients entering the analysis set, and multiple imputation of missing values. The result will be expressed as the covariate-adjusted difference in group averages, along with the two-sided 95 % confidence interval.

For the secondary outcomes, similar analyses will be used, taking into consideration the scale of the variable (e.g., logistic regression for binary outcomes). A sensitivity analysis will be based on the per protocol set for the available cases (without imputation). Linear regression and logistic regression analyses will be conducted to examine the predictive power of the different covariates (i.e., personality pathology, mentalizing, and attachment) on treatment efficacy and treatment completion.

We will use multiple imputations for missing values in the primary and secondary outcomes [97]. Separate imputation models will be used for the two treatment groups [98]. The imputation models will include the primary and secondary endpoints (baseline and follow-up) as well as the covariates of the primary statistical analysis, and therapist time in hours.

The primary analysis will be based on the pragmatic comparison of the two treatment arms and will, therefore, not take different amounts of therapist time within or across groups into account. To account for possible differences in therapist time, a sensitivity analysis will be performed on the change scores in which the obtained values will be divided by the actual patient-specific hours of therapist contact.

Mediation analyses will be carried out to test for potential mediating effects of both mentalizing and attachment. As recommended by Hayes [99], we will conduct structural equation analyses (SEM), using Preacher and Hayes [100] methods for estimating indirect and direct effects with multiple mediators. Unstable participation in the therapy is expected. A dropout will be determined by clinicians as ending therapy without agreement with the therapist.

### Ethical considerations and regulatory approval

There are no immediate ethical problems regarding this trial. Research has not identified any significant adverse effects or risks from any of the compared interventions, and we have no advance knowledge of which intervention is most efficacious. During the trial period, any adverse event will be reported.

The trial is approved by the Regional Ethics Committee of Zealand (no: SJ-371), and is registered at the Danish Data Protection Agency (no: REG-55-2014). The trial is registered under Clinical Trials.org as NCT02068326. In accordance with the CONSORT guidelines [101, 102], we will report positive, negative, and neutral findings in the trial and we have completed the SPIRIT 2013 checklist (please see Additional file 1) and figure (please see Additional file 2).

### Confidentiality

All study-related information will be stored securely at the study site. All participant information will be stored in locked file cabinets in areas with limited access. All data will be identified by a coded identification number only to maintain participant confidentiality. All records that contain names of other personal identifiers such as locator forms and informed consent forms will be stored separately from study records identified by code number.

### Protocol amendments

Any modifications to the protocol, which may affect the conduct of the study, including changes of study objectives, study design, patient population, sample sizes, or study procedures, will require a formal amendment to the protocol. Such amendments will be registered at https://clinicaltrials.gov/ct2/show/NCT02068326 and approved by the ethics committee.

## Discussion

BPD typically emerges in adolescence and the recommended primary treatment is psychotherapeutic programs. However, only recently has research begun to investigate essential questions such as the relative effectiveness of separate program components and which level of specialization versus generalist treatment is needed for treatment of adolescent BPD to be successful [103]. The evidence base for cost-effective treatment programs for adolescent BPD is limited.

This is the first randomized controlled trial to test the efficacy of a MBT in groups for adolescents with BPD or subthreshold BPD. Below, we discuss some of the potential limitations and strengths of the M-GAB trial.

The trial is subject to at least three potential limitations. First, our primary outcome measure, the BPFS-C, was selected because it reliably and validly measures borderline personality features *specifically* in adolescents (rather than in adults) and has shown to be a sensitive outcome measure for adolescents in a previous randomized trial [53]. However, although patient-reported outcomes have obvious benefits [104], they are not observer blinded and therefore at risk of being assessed with some bias. In our trial, patients allocated to MBT may be biased by their knowledge of having received a relatively specific psychotherapeutic treatment and therefore be more optimistic about their outcome. In response to this potential limitation, we included an observer rated measure of change in BPD symptoms, the ZAN-BPD [88], as a secondary outcome.

Second, no standardizations for treatment of adolescent BPD pre-existed within the child and adolescent department, Region Zealand and for the purpose of this study, the limit for the minimum treatment in the TAU arm was defined as 12 monthly individual sessions. Individual treatment allows therapists to focus more intensely on the patient compared to group-based treatment and therapists in the TAU arm may choose to deliver treatment to some patients with a higher frequency than once a month. However, this does not completely rule out the risk of a dose–response effect since MBT is delivered on a weekly basis. We defined the minimum frequency for TAU treatment in order to minimize the possible effect of dose–response, but as completely equal doses of treatment in the two treatment arms is not guaranteed, the risk is not completely ruled out. We will monitor and register both the MBT and TAU patients’ contact to the health system during the treatment period, to be able to report on any differences in the treatment doses received.

Third, the MBT therapists are trained and supervised by engaged and specialized supervisors in a specific treatment model. It is possible that, as the supervisors believe in the advantages of the MBT model, this may influence the therapist allegiance. We were aware of the potential allegiance bias when designing the trial, but wanted to ensure therapist adherence to treatment model. The alternative of having less training or less specialized supervisors increases the risk of therapists not being adherent to the treatment model, which we considered an even greater risk to the trial.

We would also like to emphasize at least three possible strengths of the trial. First, we included borderline severity as a stratification variable in order to minimize the risk of confounding our findings by an unbalanced distribution of patients with BPD at the threshold versus subthreshold level. Second, we included outcome measures relying on different methods and sources of data (self-report, parental reports, observer-rated interviews and expert ratings) and covering different aspects of quality of life such as symptomatic improvement and social functioning. By including multiple outcome measures, we hope to be able to discuss outcome in broader terms. Third, in the selection of in- and exclusion criteria we aimed for participants to be as close to patients with BPD or subthreshold BPD typically referred to treatment at Child and Adolescent Psychiatric Department, Region Zealand. For example, patients living with foster parents or other kinds of parental substitutes and patients presenting comorbidity or substance abuse were included. Accordingly, the findings of the trial should have a wide generalizability.

## Trial status

The trial is currently in the recruitment phase. The first patient was included and randomized on 24 September 2015 and inclusion is expected to be completed in January 2017.

## Abbreviations

M-GAB, Mentalization-Based Treatment in Groups for Adolescents with Borderline Personality Disorder (BPD) or subthreshold BPD versus Treatment As Usual; BDI-Y, Becks Depression Inventory for Youth; BPD, borderline personality disorder; BPFS-C, Borderline Personality Features Scale for Children; BPFS-C-11, Borderline Personality Features Scale for Children-Short version; BPFS-P, Borderline Personality Features Scale for Children-Parents’ version; CAT, cognitive analytic therapy; CBCL, Childhood Behavior Checklist; C-GAS, Children's Global Assessment Scale; CI-BPD, Childhood Interview for DSM-IV Borderline Personality Disorder; DBT, dialectical behavior therapy; DSM-5, *Diagnostic and Statistical Manual of Mental Disorders, fifth edition*; ECR, Experience of Close Relationships Inventory; IPPA-R, Inventory of Parent and Peer Attachment-Revised; ITT, intention to treat; MINI-KID, Mini International Neuropsychiatric Interview for children and adolescents; MBT, mentalization-based treatment; MBT-G, mentalization-based treatment in groups; MBT-I, mentalization-based treatment-introduction; MGT-P, mentalization-based psychoeducation therapy for parents of patients; PAI, Personality Assessment Inventory; PD, personality disorder; RCT, randomized controlled trial; RFQ-Y, Reflective Function Questionnaire for Youth; RTSHIA, Risk-Taking and Self-Harm Inventory for adolescents; SCID-II, Structured Clinical Interview for DSM-IV-Axis II; TAU, treatment as usual; YSR, The Youth Self-Report; ZAN-BPD, Zanarini Rating Scale for Borderline Personality Disorder

## Additional files

Additional file 1: SPIRIT 2013 checklist: recommended items to address in a clinical trial protocol and related documents^*^. (DOC 125 kb) Additional file 2: M-GAB SPIRIT 2013 Figure. (DOC 58 kb)
