# Supplementary material for: Mentalization-based treatment in groups for adolescents with borderline personality disorder (BPD) or subthreshold BPD versus treatment as usual (M-GAB): study protocol for a randomized controlled trial
Source: Trials. 2016 Jul 12;17:314. doi: 10.1186/s13063-016-1431-0 (PMC4942923; doi:10.1186/s13063-016-1431-0)
Supplement: Additional file 2: — M-GAB SPIRIT 2013 Figure. (DOC 58 kb) [file 13063_2016_1431_MOESM2_ESM.doc]

**M-GAB SPIRIT 2013 Figure**

|  | **STUDY PERIOD** | | | | | | | |
| --- | --- | --- | --- | --- | --- | --- | --- | --- |
|  | **Enrolment** | **Allocation** | **Post-allocation** | | | | | |
| **TIMEPOINT**** | ***Base-***  ***line*** | **0** | ***Week 10*** | ***Week***  ***20*** | ***Week***  ***30*** | ***Dis-charge*** | ***3 months follow-up*** | ***12 months follow-up*** |
| **ENROLMENT:** | X |  |  |  |  |  |  |  |
| **Eligibility screen** | X |  |  |  |  |  |  |  |
| **Informed consent** | X |  |  |  |  |  |  |  |
| **Allocation** |  | X |  |  |  |  |  |  |
| **INTERVENTIONS:** |  |  |  |  |  |  |  |  |
| ***MBT I Groups*** |  |  |  |  |  |  |  |  |
| ***Treatment as usual*** |  |  |  |  |  |  |  |  |
| **ASSESSMENTS:** |  |  |  |  |  |  |  |  |
| **SCID-II** | X |  |  |  |  |  |  |  |
| **CI-BPD** | X |  |  |  |  |  |  |  |
| **M.I.N.I.-KID** | X |  |  |  |  |  |  |  |
| **CGAS** | X |  |  |  |  | X |  |  |
| **ZAN-BPD** | X |  |  |  |  | X |  |  |
| **BPFS-C** | X |  | X | X | X | X | X | X |
| **YSR** | X |  | X | X | X | X | X | X |
| **BDI-Y** | X |  | X | X | X | X | X | X |
| **RTSHIA** | X |  | X | X | X | X | X | X |
| **ECR-R** | X |  | X | X | X | X | X | X |
| **IPPA-R** | X |  | X | X | X | X | X | X |
| ***RFQ-Y*** | X |  | X | X | X | X | X | X |
| **Parent: CBCL** | X |  | X | X | X | X | X | X |
| **Parent: BPFS-P** | X |  | X | X | X | X | X | X |
